# Correlation between Hearing Impairment and the Triglyceride Glucose Index in Middle-Aged Female Based on a Korean National Health and Nutrition Examination Survey

**DOI:** 10.3390/medicina60101596

**Published:** 2024-09-28

**Authors:** Dong Oh Kim, Youngin Lee, Sang Yeoup Lee, Jeong Gyu Lee, Yu Hyeon Yi, Young Hye Cho, Young Jin Tak, Eun Ju Park, Seung Hun Lee, Gyu Lee Kim, Jung In Choi, Young Jin Ra, Sae Rom Lee, Ryuk Jun Kwon, Soo Min Son, Su Min Lee, Jong Suk Lee

**Affiliations:** 1 Department of Family Medicine and Research Institute for Convergence of Biomedical Science and Technology, Pusan National University Yangsan Hospital, Yangsan 50612, Republic of Korea; kdoh2002@naver.com (D.O.K.);; 2 Department of Family Medicine, Pusan National University School of Medicine, Yangsan 50612, Republic of Korea; 3 Department of Medical Education, Pusan National University School of Medicine, Yangsan 50612, Republic of Korea; 4 Department of Family Medicine, Pusan National University Hospital, Busan 49241, Republic of Korea

**Keywords:** hearing loss, insulin resistance, TyG index, middle-aged female, diabetes

## Abstract

*Background and Objectives*: This study aimed to investigate the association between insulin resistance, as measured by the triglyceride–glucose index (TyG index), and hearing impairment in middle-aged women in Korea. *Materials and Methods*: This cross-sectional survey utilized data from the Korea National Health and Nutrition Examination Survey (KNHANES) IV (2007–2009), specifically from the period after July 21, 2009, when hearing test results became available, and from the KNHANES V (2010–2012). This study was conducted on 5416 women aged 40 to 69 who had completed both the health examination survey and audiometric tests, excluding those with missing data on menopausal status and the use of hormone replacement therapy. *Results*: In the study group, the prevalence of high-frequency hearing loss according to the TyG index was significantly higher in the mild hearing loss group (OR = 1.29; 95% CI: 1.12, 1.49, *p* < 0.001) and the moderate hearing loss group (OR = 1.27; 95% CI: 1.09, 1.48, *p* = 0.002). Conversely, the prevalence of low-frequency hearing loss did not show a significant difference in either the mild hearing loss group (OR = 1.17; 95% CI: 0.99, 1.37, *p* = 0.065) and the moderate hearing loss group (OR = 1.13; 95% CI: 0.94, 1.35, *p* = 0.199) *Conclusions*: Since diabetes can induce hearing impairment in women, it is recommended that women with a high TyG index undergo early hearing tests

## 1. Introduction

Hearing impairment is one of the major disabilities [1] yet hearing loss screenings are not properly conducted in daily life. Many people are unaware of the need for hearing screenings and believe that the cost is high. This makes it difficult to administer timely treatment [2].

There are various causes of hearing loss, including presbycusis, noise exposure, ototoxic drugs, and viral infections [3]. Presbycusis is a common chronic condition among the elderly, and diabetes has been reported to exacerbate age-related hearing loss [4] and is also recognized as an independent factor for hearing loss regardless of age [5].

Diabetes can cause vascular diseases and neurological complications in the long term [6]. Recent studies have suggested that hearing loss is a new complication of diabetes. Numerous studies and large-scale meta-analyses have found a significant association between diabetes and sensorineural hearing loss [5,6,7,8,9].

The mechanisms by which diabetes induces hearing loss include microangiopathy, advanced glycation end products, and oxidative stress pathways [10]. Although the exact mechanisms are still unclear, hyperglycemia is known to damage the microvascular structure of the inner ear tissues, impair oxygen supply, and cause demyelination of the auditory nerve and loss of spiral ganglion cells and outer hair cells [11,12]. HOMA-IR is used to evaluate insulin resistance in diabetes, and there are studies investigating the association between HOMA-IR, impaired fasting glucose (IFG), and hearing loss [13]. While HOMA-IR is widely used as a non-invasive method to study insulin resistance, its value diminishes in patients undergoing insulin treatment or those lacking functional β-cells [14]. Therefore, there is a need for new methods to assess insulin resistance, and the TyG index can be used instead of HOMA-IR [15]. Research has shown that the TyG index can more robustly predict various complications such as cardiovascular diseases [16], type 2 diabetes [17], and polycystic ovary syndrome [18], and it is easier to apply in clinical practice [19]. Due to insulin resistance primarily affecting high-frequency hearing loss [13], this study also distinguishes between high-frequency and low-frequency hearing loss. Additionally, there are studies on the correlation between the TyG index and hearing loss, but these studies have the limitation of being based on the NHANES cohort, making it difficult to apply their findings to the Korean population [20].

Estrogen has been found to influence hearing protection positively, whereas progestin has a negative impact on hearing [21]. There are also studies suggesting that hormone replacement therapy helps reduce hearing loss [22]. Therefore, in studies on hearing loss in women, it is necessary to account for menopause status and hormone replacement therapy use. However, to date, no studies have adjusted for hormone replacement therapy when examining the association between the TyG index and hearing loss. This study investigated the association between the TyG index and hearing loss in a representative sample of Korean women aged 40–69, adjusting for menopause status and hormone replacement therapy use.

## 2. Materials and Methods

### 2.1. Study Subjects

This cross-sectional survey utilized data from the Korea National Health and Nutrition Examination Survey (KNHANES) IV (2007–2009), which has been conducted periodically by the Ministry of Health and Welfare of South Korea since 1998. Specifically, this study utilized data from July 21, 2009, when hearing test results became available, and from the KNHANES V (2010–2012). A total of 36,067 individuals completed the survey during this period. This study focused on women aged 40–69 years, excluding those with missing data on menopause status and hormone replacement therapy usage, and included only those who completed both the health examination survey and audiometric test (*n* = 5416). The survey design was reviewed by the Institutional Review Board of the Korea Centers for Disease Control and Prevention (2009-01CON-03-2C, 2010-02CON-21-C, 2011-02CON-06-C, 2012-01EXP-01-2C).

### 2.2. Laboratory Tests

During the survey, overnight fasting blood samples were collected from all participants in the morning. These samples were immediately refrigerated and transported to a central testing laboratory in Seoul, South Korea. All blood samples were analyzed within 24 h of transportation. Fasting triglyceride and fasting glucose levels were measured, and the TyG index was calculated using the following formula:

$$\ln\begin{array}{c} \left[fasting\;triglycerides\left(\frac{mg}{dL}\right)\times FBG\left(\frac{mg}{dL}\right)÷2\right] \end{array}.$$

### 2.3. Audiometric Tests

Trained otolaryngologists conducted pure-tone audiometry at six frequencies (500, 1000, 2000, 3000, 4000, and 6000 Hz) for each ear using a GSI SA-203 automatic audiometer manufactured by Entomed Diagnostics AB, Malmö, Sweden, in a soundproof booth. Hearing impairment was classified into low-/high-frequency and mild/moderate categories. The low-frequency pure-tone average (PTA) was defined as the mean hearing threshold at 500, 1000, and 2000 Hz for each ear. The high-frequency PTA was defined as the mean hearing threshold at 3000, 4000, and 6000 Hz for each ear. Mild hearing impairment was defined as an unaided pure-tone average of 26–40 dB in the better ear. Moderate hearing impairment was defined as an unaided pure-tone average of ≥40 dB in the better ear.

### 2.4. Prevalence of Hearing Loss

The prevalence of hearing loss was calculated as the point prevalence based on data from the Korean National Health and Nutrition Examination Survey (KNHANES). It was determined as the proportion of participants who met the hearing impairment criteria described in Section 2.3 among the participants targeted in this study, as described in Section 2.1 (*n* = 5416).

### 2.5. Statistical Analysis

The distribution of demographic, socioeconomic, and clinical characteristics was analyzed using the chi-squared test and one-way ANOVA. The association between the TyG index and the prevalence of hearing impairment was assessed using multivariable logistic regression analysis. After adjusting for menopausal status, hormone replacement therapy use, smoking, income, education level, and alcohol consumption, odds ratios (ORs) and 95% confidence intervals (CIs) were calculated. Statistical analyses were performed using SPSS version 27.0.

## 3. Results

The basic characteristics of the study population according to the presence of hearing loss are shown in Table 1 and Table 2. A total of 5416 female participants aged 40 to 69 years were selected, and the proportions of normal hearing, mild hearing loss, and severe hearing loss in the high-frequency range were presented according to various variables. The mean ages for the normal, mild hearing loss, and moderate hearing loss groups were 48.70 ± 0.16, 54.72 ± 0.25, and 57.77 ± 0.29, respectively. Characteristics affecting high-frequency hearing loss included age, alcohol consumption, income level, education level, menopause status, and use of female hormones (*p* < 0.01). Smoking did not influence high-frequency hearing loss (*p* = 0.297).

Characteristics influencing low-frequency hearing loss included age, alcohol consumption, income level, education level, and menopause status (*p* < 0.01). Smoking (*p* = 0.893) and hormone replacement therapy (*p* = 0.21) did not affect low-frequency hearing loss.

### 3.1. Prevalence of Hearing Loss According to the TyG Index

The prevalence of high-frequency hearing loss increased with higher TyG index values. As shown in Table 3, in the crude model, high-frequency hearing loss was observed in the mild group (OR = 1.38; 95% CI: 1.38, 1.83, *p* < 0.001) and the moderate group (OR = 1.68; 95% CI: 1.46, 1.94, *p* < 0.001).

The prevalence of low-frequency hearing loss increased with higher index values. As shown in Table 4, in the crude model, low-frequency hearing loss was observed in the mild hearing loss group (OR = 1.44; 95% CI: 1.23, 1.67, *p* < 0.001) and in the moderate hearing loss group (OR = 1.38; 95% CI: 1.16, 1.64, *p* < 0.001).

### 3.2. Prevalence of Hearing Loss According to the TyG Index after Adjusting for Variables

In Model 2, after adjusting for menopausal status, hormone replacement therapy use, smoking, income, education level, and alcohol consumption, a higher TyG index was associated with a significantly increased risk of high-frequency hearing loss. Specifically, participants with higher TyG index values had a higher prevalence of high-frequency hearing loss, both in the mild hearing loss group (OR = 1.29; 95% CI: 1.12, 1.49, *p* < 0.001) and the moderate hearing loss group (OR = 1.27; 95% CI: 1.09, 1.48, *p* = 0.002).

Conversely, the association between the TyG index and low-frequency hearing loss was not significant in either the mild hearing loss group (OR = 1.17; 95% CI: 0.99, 1.37, *p* = 0.065) and the moderate hearing loss group (OR = 1.13; 95% CI: 0.94, 1.35, *p* = 0.199).

## 4. Discussion

In this study, a high TyG index is an independent risk factor for hearing impairment in Korean women aged 40–69, even after adjusting for several variables. Diabetes mellitus is known to be associated with hearing impairment, and the TyG index may be used to predict diabetes. Previous studies have investigated the impact of IFG and diabetes on hearing impairment using HOMA-IR in a Korean population [13]. In that study, significant associations between IFG, HOMA-IR, HOMA-b, and hearing impairment were observed only in men, consistent with prior research [8,23]. On the other hand, this study suggests that insulin resistance, as measured by the TyG index, may be associated with high-frequency hearing loss in women, after adjusting for menopausal status and hormone replacement therapy.

Although diabetes mellitus is a well-established risk factor for hearing impairment, the exact mechanisms through which diabetes induces hearing impairment remain unclear [24]. Microvascular disease and peripheral neuropathy resulting from diabetes have been suggested as contributing factors to hearing impairment [25,26]. One study reported that auditory dysfunction related to peripheral neuropathy is mainly associated with chronic hyperglycemia rather than acute hyperglycemia [27]. This highlights the potential significance of the TyG index in patients with diabetes. Studies testing insulin resistance using the TyG index have demonstrated that it is superior to the HOMA-IR index due to its insulin independence [19,28,29].

Previous research has indicated that estradiol has a protective effect on hearing [30,31], but some studies [32] have reported that oral estradiol increases the risk of hearing loss in postmenopausal women, with the duration of oral estradiol use being significantly associated with the risk of hearing impairment. Meanwhile, nearly all researchers agree that progesterone can cause hearing loss [32,33], being linked to increased vascular inflammation in the inner ear [34].

The strengths of this study are its focus on a representative sample of the general female population in Korea and a relatively large sample size. However, this study has some limitations. First, as this study is cross-sectional, this study does not fully establish causality between insulin resistance and hearing loss in women. Additional longitudinal studies are required to verify this. Second, there are missing data on menopausal status and hormone therapy use in the KNHANES dataset, and there was no survey on the types, dosage, and duration of hormone therapy use. Further research should control for the types, dosage, and duration of hormone therapy use.

Age-related hearing loss can begin as early as 40–50 years old, initially affecting high-frequency hearing and subsequently impacting low-frequency hearing [35]. Diabetic patients under 60 years old show early high-frequency hearing impairment, and the difference in hearing loss between diabetic and non-diabetic individuals decreases thereafter [36].

## 5. Conclusions

Since diabetes can induce hearing impairment in women, it is recommended that early hearing tests be conducted for women with a high TyG index to screen for potential hearing loss.

## Figures and Tables

**Table 1:** General characteristics of study subjects with high-frequency hearing loss.

		Normal	Mild	Moderate	
Total no. subjects		2652	1495	1269	
Variable	Category	M ± SE/n(%)	M ± SE/n(%)	M ± SE/n(%)	*p*
Age	Year	48.70 ± 0.16	54.72 ± 0.25	57.77 ± 0.29	<0.001
Smoking	Less than 5P	26(1.2)	12(0.6)	13(1.4)	0.297
	More than 5P	155(6.8)	102(8.1)	66(6.3)	
	No smoking	2471(92.0)	1381(91.3)	1190(92.3)	
Drinking (1Y)	No (1Y)	442(16.0)	239(15.8)	213(17.3)	<0.001
	Less than 1 in Month	746(28.8)	399(27.6)	332(26.0)	
	1 in month	358(13.6)	149(10.2)	128(11.1)	
	2–4 in month	458(17.7)	223(15.5)	157(14.5)	
	2–3 in week	159(6.5)	85(7.3)	44(4.5)	
	More than 4 in week	52(2.2)	23(1.9)	24(1.8)	
	No drinking	437(15.4)	377(21.7)	371(24.8)	
Income	Lower	253(9.7)	317(18.6)	379(26.9)	<0.001
	Lower-middle	613(25.4)	405(27.7)	380(28.7)	
	Upper-middle	775(29.6)	393(27.6)	271(24.2)	
	Upper	1011(35.3)	380(26.1)	239(20.2)	
Education level	Elementary school or below	326(11.3)	473(29.0)	580(42.6)	<0.001
	Highschool graduate or below	1769(69.0)	906(64.0)	632(53.1)	
	College graduate or above	557(19.8)	116(7.0)	57(4.3)	
Menopausal status	No	1625(67.0)	512(40.1)	365(34.8)	<0.001
	Yes	1027(33.0)	983(59.9)	904(65.2)	
Hormone replacement therapy	No	292(9.3)	263(14.9)	234(15.9)	<0.001
	Yes	2360(90.7)	1232(85.1)	1035(84.1)	
BMI		23.80 ± 0.08	24.34 ± 0.13	24.26 ± 0.11	<0.001
SBP		116.86 ± 0.40	121.35 ± 0.57	124.08 ± 0.70	<0.001
DBP		75.99 ± 0.24	77.02 ± 0.32	76.90 ± 0.38	0.011
Total cholesterol		194.45 ± 0.83	198.39 ± 1.04	199.77 ± 1.34	<0.001
HDL		52.21 ± 0.27	50.01 ± 0.37	49.29 ± 0.39	<0.001
Triglyceride		114.07 ± 2.36	133.02 ± 2.79	133.98 ± 3.06	<0.001
eGFR		101.08 ± 0.30	97.09 ± 0.38	95.50 ± 0.47	<0.001
Hypertension	Yes	416(14.2)	413(24.4)	461(34.1)	<0.001
Hyperlipidemia	Yes	342(11.2)	264(15.2)	245(17.5)	<0.001
TyG_Index		8.44 ± 0.01	8.61 ± 0.02	8.63 ± 0.02	<0.001

Values are presented as mean ± standard deviation or number (%).

**Table 2:** General characteristics of study subjects with low-frequency hearing loss.

		Normal	Mild	Moderate	
Total no. subjects		4048	845	523	
Variable	Category	M ± SE/n(%)	M ± SE/n(%)	M ± SE/n(%)	*p*
Age	Year	50.70 ± 0.15	57.09 ± 0.37	57.19 ± 0.46	<0.001
Smoking	Less than 5P	40(1.2)	7(0.8)	4(0.9)	0.893
	More than 5P	241(7.0)	51(7.7)	31(6.9)	
	No smoking	3767(91.9)	787(91.6)	488(92.2)	
Drinking (1Y)	No (1Y)	663(15.8)	134(16.8)	97(19.0)	<0.001
	Less than 1 in Month	1139(29.0)	221(25.1)	117(22.5)	
	1 in month	495(12.4)	86(11.8)	54(10.6)	
	2–4 in month	662(17.0)	107(14.2)	69(14.7)	
	2–3 in week	235(6.8)	31(4.5)	22(4.9)	
	More than 4 in week	68(1.9)	20(2.2)	11(2.7)	
	No drinking	786(17.1)	246(25.4)	153(25.6)	
Income	Lower	558(12.7)	237(25.5)	154(25.0)	<0.001
	Lower-middle	1006(26.1)	248(30.4)	144(26.7)	
	Upper-middle	1140(29.2)	178(21.4)	121(27.2)	
	Upper	1344(32.0)	182(22.7)	104(21.1)	
Education level	Elementary school or below	791(17.1)	368(41.5)	220(39.4)	<0.001
	Highschool graduate or below	2602(67.4)	425(52.3)	280(56.7)	
	College graduate or above	655(15.5)	52(6.3)	23(3.8)	
Menopausal status	No	2080(58.1)	242(35.3)	180(38.7)	<0.001
	Yes	1968(41.9)	603(64.7)	343(61.3)	
Hormone replacement therapy	No	547(11.4)	148(14.1)	94(15.5)	0.021
	Yes	3501(88.6)	697(85.9)	429(84.5)	
BMI		23.99 ± 0.07	24.24 ± 0.14	24.13 ± 0.17	0.125
SBP		118.51 ± 0.35	122.11 ± 0.79	124.41 ± 1.13	<0.001
DBP		76.44 ± 0.21	76.21 ± 0.43	76.88 ± 0.56	0.510
Total cholesterol		196.01 ± 0.67	198.30 ± 1.49	198.89 ± 1.79	0.091
HDL		51.50 ± 0.22	48.90 ± 0.49	50.18 ± 0.63	<0.001
Triglyceride		120.40 ± 1.88	133.43 ± 3.58	131.40 ± 4.38	<0.001
eGFR		99.73 ± 0.26	95.66 ± 0.58	96.43 ± 0.63	<0.001
Hypertension	Yes	807(17.5)	286(31.0)	197(35.3)	<0.001
Hyperlipidemia	Yes	573(12.4)	176(17.6)	102(17.4)	0.001
TyG_Index		8.49 ± 0.01	8.63 ± 0.03	8.62 ± 0.03	<0.001

**Table 3:** High-frequency hearing loss according to the TyG index.

HFHL (Ref: Normal)	Crude Model			Adjusted Model 1			Adjusted Model 2		
	OR	95% CI	*p*	OR	95% CI	*p*	OR	95% CI	*p*
Mild	1.59	1.38, 1.83	<0.001	1.41	1.22, 1.62	<0.001	1.29	1.12, 1.49	<0.001
Moderate	1.68	1.46, 1.94	<0.001	1.46	1.26, 1.69	<0.001	1.27	1.09, 1.48	0.002

Adjusted Model 1: menopausal status and hormone replacement therapy; Adjusted Model 2: model 1 + smoking, income, education level, and alcohol consumption.

**Table 4:** Low-frequency hearing loss according to the TyG index.

LFHL (Ref: Normal)	Crude Model			Adjusted Model 1			Adjusted Model 2		
	OR	95% CI	*p*	OR	95% CI	*p*	OR	95% CI	*p*
Mild	1.44	1.23, 1.67	<0.001	1.29	1.10, 1.51	0.002	1.17	0.99, 1.37	0.065
Moderate	1.38	1.16, 1.64	<0.001	1.26	1.06, 1.49	0.010	1.13	0.94, 1.35	0.199

Adjusted model 1: menopausal status and hormone replacement therapy; Adjusted Model 2: model 1 + smoking, income, education level, and alcohol consumption.

## Data Availability

Data and materials are available upon reasonable request. The raw KNHANES data used in this paper can be accessed via the following website: https://knhanes.kdca.go.kr/knhanes/sub03/sub03_02_05.do (accessed on 15 August 2024).
